# Visual perception from the perspective of a representational, non-reductionistic, level-dependent account of perception and conscious awareness

**DOI:** 10.1098/rstb.2013.0209

**Published:** 2014-05-05

**Authors:** Morten Overgaard, Jesper Mogensen

**Affiliations:** 1 CNRU, CFIN, MindLab, Aarhus University, Noerrebrogade 44, Building 10G, DK-8000 Aarhus, Denmark; 2 CCN, Department of Psychology and Communication, Aalborg University, Aalborg, Denmark; 3 Unit for Cognitive Neuroscience (UCN), Department of Psychology, University of Copenhagen, Copenhagen, Denmark

**Keywords:** consciousness, brain, blindsight, unconscious, perception, neuroscience

## Abstract

This article proposes a new model to interpret seemingly conflicting evidence concerning the correlation of consciousness and neural processes. Based on an analysis of research of blindsight and subliminal perception, the reorganization of elementary functions and consciousness framework suggests that mental representations consist of functions at several different levels of analysis, including truly localized perceptual elementary functions and perceptual algorithmic modules, which are interconnections of the elementary functions. We suggest that conscious content relates to the ‘top level’ of analysis in a ‘situational algorithmic strategy’ that reflects the general state of an individual. We argue that conscious experience is intrinsically related to representations that are available to guide behaviour. From this perspective, we find that blindsight and subliminal perception can be explained partly by too coarse-grained methodology, and partly by top-down enhancing of representations that normally would not be relevant to action.

## 1. Introduction

We still have no generally accepted or comprehensive understanding of the mind–brain problem. Human consciousness can be defined as inner subjective experiences, such as perceptions, judgements, thoughts, intentions to act, feelings and desires. These experiences can be described from a subjective, phenomenal first-person perspective. On the other hand, cognitive neuroscience explores the neural correlates with respect to brain topology and brain dynamics from an objective third-person perspective. This difference in perspectives is on many accounts a fundamental part of the reason why consciousness seems incompatible with neuroscience.

This paper will present a new look at how one may conceive of the relationship between conscious experience and neural processes. It will take as a point of departure an analysis of ‘blindsight’, partly because the phenomenon has had a big impact on already existing mind–brain models, and partly because research on the topic holds inconsistencies, the resolution of which may be crucial to such a theory.

Many existing models have explicitly attempted a ‘creationist’ strategy, trying to explain how consciousness arises from or is created by brain processes. As often pointed out (e.g. [1]), it is very difficult, if not impossible, to show that a particular relationship between neural and mental states indicates that the one level of description explains the other, let alone causes it. To do so, one needs a further claim saying more than just two observations go together.

The reason for our pessimism for the ‘creationist’ strategy is that we believe that such a ‘further claim’ can never be built on the same scientific grounds as the correlation. This is a consequence of the fact that the use of one level of description, referring to conscious experience, is a necessary methodological requirement to find any correlation [2]. Deducing the components of this level of description from components of another (e.g. neural phenomena) that are already mapped to the first level is obviously circular. Excluding either the correlations or descriptions of conscious states would be logically impossible as the neural phenomena (e.g. ‘brain areas’) are only ‘components’ because they are identified by virtue of their correlations with particular experiences.

These convictions are, however, not the end but rather the starting point of our analysis—leading to a framework to investigate and possibly understand consciousness in relation to neural processes.

## 2. Blindsight

The concept ‘blindsight’ refers to a ‘visual capacity in the absence of acknowledged awareness’ in relation to a lesion to the primary visual cortex [3]. As blindsight is typically conceived, it is, from the perspective of subjective experience, identical to cortical blindness or hemianopia. However, from a cognitive perspective it is quite different, as certain visual functions are preserved. It seems less important which exact functions are preserved, it is rather the very segregation of functional and phenomenal aspects of mental states that is central to the idea.

In possibly the first experimental study on blindsight (see, however [4,5]), Weiskrantz *et al*. [6] reported experiments with the now famous patient DB. DB was asked to shift his eyes from a fixation point to the position where he would guess a light was flashed. The experiment showed a weak correspondence between target position and eye movement. In a second experiment, DB now had to reach for the target with a finger instead of relying on eye movements alone. With this different method, results showed a very clear correspondence between target and finger position, especially for larger stimuli. Further experiments studied DB's ability to discriminate between two possible stimuli (X versus O, horizontal versus vertical lines, etc.) and found that he was able to do so well above chance level with increased performance as a function of stimulus size.

Many experiments have demonstrated how blindsight patients seem able to do complicated tasks in the reported absence of conscious experience, but also the limits of the visual abilities. Kentridge *et al*. [7] found that patient GY's attention could be directed by cues in the residual part of the visual field as well as in the blind field. Cues in the blind field could even direct his attention to locations in the healthy field, from which the authors conclude that spatial information selection and conscious experience rely on different processes, and that blindsight patients may have fully or partially intact visual attention without visual experience. It is repeatedly found that blindsight differs from normal vision by needing cueing and prompting for the patient to react to the visual information [8].

This finding is not specific for blindsight, but is generally the case for experiments on unconscious vision in healthy subjects. Many psychological experiments have attempted to prove the existence of unconscious perceptual processes by demonstrating that stimuli are perceived when subjects are not consciously aware of the stimuli. The basic strategy in these studies is to establish conditions under which conscious experience does not occur and then to demonstrate that stimuli can nevertheless be perceived under these conditions. The success of these studies depends crucially on two factors: first, the acceptability of the method used to establish the absence of conscious perception, and second, the method of assessing that the not consciously experienced stimulus is indeed unconsciously perceived. Today, research on unconscious vision in healthy subjects and blindsight alike is conducted using a combination of performance measures and introspective reports. Unconscious perception is defined as a successful performance (e.g. discrimination) in the absence of positive introspective reports. Studies based on both types of measures have not led to completely convincing results, because it is always possible to question whether the measure of conscious perception was successful in guaranteeing a complete absence of all relevant conscious experiences, as absence of evidence is no evidence of absence [9]. Nevertheless, the majority of cognitive scientists today seem to share the conviction that perception comes in two varieties: conscious and unconscious.

## 3. Unconsciousness or vague experiences?

One historically difficult issue for blindsight has been the fact that all or most blindsight patients have reported some vague experiences associated with the visual stimuli about which they are asked to report [10,11]. Many blindsight researchers handle such reports by subdividing blindsight into ‘type 1’ and ‘type 2’, where ‘type 1’ are patients without any conscious experiences related to visual stimuli, whereas ‘type 2’ are patients with conscious experiences related to visual stimuli. Such experiences are, however, not conceived as being of a visual nature [12].

Other experiments seem, however, to support a different perspective. Overgaard *et al*. [13] performed a study on a 31-year-old hemianopic patient with left-sided injury to primary visual cortex (GR). In the first experiment, letters were briefly flashed at different locations on a computer screen and GR's only task was to respond to every stimulus, revealing that she was blind to everything presented in the upper right quadrant. In the second experiment, GR was presented with different visual figures and asked (1) which figures were shown, and (2) if she actually saw the figure—yes or no. GR reported only very rarely that she saw stimuli in the upper right quadrant, yet she was able to report correctly about these stimuli more often than chance. In the healthy part, her reports were significant predictors of correctness, based on which the authors concluded that she had blindsight. The third experiment was identical to the second, except she now should respond with the perceptual awareness scale (PAS) with four labelled points, rather than in a binary fashion. As a consequence, her blindsight seemingly ‘disappeared’ in the sense that, even though she reported much more vague experiences in the upper right compared with the upper left quadrant, the relationship between correctness and reported experience was identical to what was found within the rest of the visual field. All correctness above chance seemed related to vague yet conscious vision when using PAS. So, the first experiment indicates that GR is a ‘cortically blind hemianopic’, the second indicates that she has blindsight, perhaps type 1, and the third that she has blindsight type 2 if blindsight at all. One might be tempted to conclude that blindsight is indeed closely related to the methods used to study conscious perception. This method-dependency is far from being a unique feature of blindsight. Within numerous cognitive domains (irrespectively of their relationship to consciousness), studies in animal models as well as humans have demonstrated that radically different conclusions of what is preserved or lost after injury to the brain depend heavily on the testing methods and experimental procedures applied (e.g. [14,15]). We will return to this and other similarities between findings within blindsight and problem solving after brain injury, respectively, in §5.

The findings obtained with GR are closely related to experiments by Stoerig & Barth [16] investigating GY, one of the most thoroughly studied blindsight patients. GY was asked to match a visual stimulus presented to the blind field with one of different image transformation of the same stimulus in the healthy field. With moving stimuli, GY accepted the match as long as they were sufficiently blurred. The results match with GY's verbal descriptions of his ‘feelings’ as ‘similar’ to that of a normally sighted man who, with his eyes shut against sunlight, can perceive the direction of motion of a hand waved in front of him.

The conclusion that the method for reporting has important consequences for findings of unconscious perception has been reached many times with healthy subjects. The PAS was first developed by Ramsøy & Overgaard [17], who asked subjects to create their own scale for subjective reporting with the instruction that they should be able to subjectively experience the difference between the scale points they invented. Most subjects conformed to a four-point scale categorized as ‘not seen’, ‘weak glimpse’ (meaning ‘something was there but I had no idea what it was’), ‘almost clear image’ and ‘clear image’. Ramsøy and Overgaard showed that in an experimental design where one should expect to find subliminal perception, subjects were completely at base chance when reporting ‘not seen’. In a later study, dichotomous reports were compared directly with PAS [18]. Using the dichotomous report, subjects showed subliminal perception, whereas none was present at PAS = ‘not seen’. In further studies, PAS was found to be more sensitive to subjective experience than other four-point scales [19]. Generally, PAS fitted better with objective measures such as stimulus duration and correctness than did the dichotomous report.

## 4. Mental states, representation and gradedness

Much conceptual work has attempted to make clear distinctions between different notions of mental states. Ned Block [20] has famously distinguished between phenomenal consciousness (‘P-consciousness’) and access consciousness (‘A-consciousness’). P-consciousness denotes subjective experience, such as the taste of coffee, seeing the colour red or thinking about a sunny day. A-consciousness denotes states on the basis of which a subject is able to reason, and to have rational control of action and speech. Hypothetically, the two meanings of the term give rise to two meanings of unconsciousness as well, namely ‘P-unconscious’ (yet potentially still A-conscious) and ‘A-unconscious’ (yet still P-conscious). The latter of the two ideas is the most controversial, and to many scientists it is conceptual only. The first idea, A without P, is, however, the important case in blindsight. If occipital lobe injured patients are able to perform certain judgements based on visual stimuli without reports about having experienced those stimuli, this may satisfy this idea.

However, experiments such as the ones described above challenge the idea that mental states come as ‘on’- or ‘off’-versions of states with otherwise identical contents. Instead, they suggest a graded account with many stages or ‘levels’. This is, however, not at all an unusual situation in neuropsychology. When one scrutinizes clinical results, patients who lose particular functions or abilities as a consequence of brain injury rarely lose all aspects of those functions, and rarely lose accompanying experiences as well. Patients with working memory dysfunctions after neural injury do not lose working memory altogether [21]. Patients with prosopagnosia after, typically, injury to the fusiform gyrus, do not experience ‘nothing’ where there should have been a face, but rather ‘something unfamiliar’ [22]. In other words, it is, in a way, already well-established that many cognitive functions come in degrees. Still, however, the ‘on–off’ interpretation of mental states is often reflected in theories about the neural correlate of consciousness that look for particular areas or processes in the brain that are associated with the ‘on’ state (e.g. [23]). This type of approach considers particular areas or brain processes as ‘add-ons’ to the ‘off’ versions of the identical mental states, and therefore seems related to the notion of ‘consciousness arising’ from those particular brain processes.

Recent theoretical developments, such as the ‘partial awareness hypothesis’ offer alternative ideas [24]. Here, it is argued that ‘access’, as in Block's A-consciousness, should be understood as a hierarchy of representational levels where each level may be accessed individually. Our proposal shares some of the basic assumptions with the partial awareness hypothesis, e.g. that information in the brain is processed in a hierarchical manner from very simple features of, say, visual objects (e.g. flickers of light, contours or movements) to complex conglomerates of information (e.g. meaning or recognition). Here, however, we take this idea a step further and argue that the representational account of mental states, conscious experience and action stand in a necessary, yet plastic and dynamic, mutually fixed relationship.

All experiments reviewed above suggest that patients as well as healthy individuals perform better in various tasks when reporting experiences of relevant objects of the task than when they do not. The experiments using PAS even suggest almost perfect correlations between performance and clarity of experience where difficulties could be described as a ‘lag’ between two sigmoid curves rather than as fundamentally different categories [25,26]. On the basis of evidence, we propose that there is an intrinsic relationship of some kind between a functional account of mental representation and conscious experience, so that the more elaborate a mental representation is, the more clearly the particular object is perceived. In conceptual terms, the proposal goes against the idea that a conceptual division between ‘A-’ and ‘P-consciousness’ reflects an empirical division.

By ‘intrinsic relationship’ we wish to express that we believe this correlational relationship reflects some more fundamental, or ontological, relationship. However, for reasons stated above, we do not believe that the relationship between functional and phenomenal aspects of mind, as well as the relationship between mind and brain, can be expressed as identity or in terms of reduction. Our choice of words—that the relation is ‘intrinsic’—relates to David Chalmers’ proposal that some (yet undiscovered) natural law connects conscious experience with information (*per se*) [27]. In our version, there is, obviously, no evidence for a connection between experience and ‘information’, in this very broad sense, but rather for a relationship between experience and at least one very specific kind of information: representations that are available for action, e.g. a report.

Blindsight fits well into this conception. Whereas some experiments that look for relationships between performance and graded experience find some level of experience underlying imperfect yet above chance performance [13,16,28], other experiments that do not look for gradedness find good performance when patients tend to report no experiences (e.g. [29]). However, almost all blindsight experiments, as well as experiments on unconscious perception in healthy subjects, make use of prompting, forced choice guessing and no information irrelevant for the experiment competing for attention. As mentioned, some experiments have shown that very weak experiences of ‘something being present’ correlate with a not perfect but above chance performance. The same blindsight patients seem unable to use such weak experiences in everyday life, or even in more standard neuropsychological testing, where they appear completely blind. Together, these findings indicate that strong, top-down functions should be considered as part of a neuroscientific model of consciousness where information that typically is of little or no use as a guide for action can be enhanced and selected as a basis for report under such prompting conditions. Such top-down ‘enhancement’ related to specific tasks or instructions have been shown in cognitive training experiments with visual stimuli (e.g. [30]).

Arguably, our reasoning here indicates that the relationship between experience and action is so that the information that is most available and relevant to action is what is experienced most clearly. From this perspective, top-down functions make it possible to enhance the analysis or at least availability of specific over other information. In this way, the top-down selection of particular representations allows for, say, visual search in complex scenes or even searching in one's own memories of past events. However, in most normal cases, information that is represented with most detail and richness allows more flexible behaviour without any need for forced choice options. We suggest that this explains the findings that have led to ‘global workspace’ models of consciousness (e.g. [31]).

## 5. Representations as strategies

Whereas we believe that our proposal can account for all available experimental data and, at the same time, of at least our own introspective accounts of what kind of information is experienced, an important aspect of the review in §§2 and 3 has so far been overlooked. An intrinsic relationship between representation and experience, modulated by relevance to action, does not in and of itself explain why experiments in blindsight and animal models reveal that results about what is preserved after injury depend on the kind of task or testing method that is applied.

Experiments on blindsight have revealed more than whether it represents conscious or unconscious vision (or, as it seems, something more graded in between). Some experiments also give indications of whether the contents of weak experiences in blindsight are different from contents in normal vision. Azzopardi & Cowey [32] performed a signal detection analysis on GY's yes/no detection judgements and forced choice detection tasks, and found that his sensitivity was significantly higher during the forced choice task. This is different from the performance of healthy subjects when having ‘near-threshold vision’, indicating that visual stimuli in blindsight are processed ‘in an unusual way’ [32; p. 14 190].

In another study, Morland *et al*. [33] demonstrated that the famous blindsight patient GY is able to match some but not all aspects of visual stimuli when asked to compare stimuli presented to the blind and healthy hemifields. In the experiment, GY associated colour and motion correctly, but not brightness, based on which the authors argue for an anatomical separation of these functions. Morland *et al*. suggest the results indicate that GY has visual awareness of those stimulus properties for which he gave correct reports, yet, as they somewhat ambiguously put it, he is not visually aware owing to lack of or limited processing of brightness.

In sum, research on blindsight has so far generated results similar to certain central aspects of research on ‘healthy unconscious vision’, and, at the same time, that are quite different in other aspects.

This situation bears resemblance to results obtained in studies addressing the neural and cognitive mechanisms of problem solving in brain-injured and post-traumatically recovered individuals. Such mechanisms have been most thoroughly addressed in studies using animal models (e.g. [14,34–36]). In some instances, post-traumatic rehabilitative training of a cognitive task can lead to a solution equally proficient to that seen in intact individuals [37]. Apparently, the experimental animals lacking, e.g. the hippocampus or the prefrontal cortex, are nevertheless able to achieve a task solution similar to that seen in intact individuals.

In these cases, the proficiency of recovered, injured subjects and normal individuals, respectively, is similar. However, a conclusion regarding identical mechanisms of task solution does not withstand further scrutiny. Subjecting the animals to organic and behavioural/cognitive ‘challenges’ (see [14,36]) allows a more thorough analysis of the neural and cognitive mechanisms mediating the task solution. Even when normal and recovered brain-injured individuals have achieved identical levels of solution proficiency, such an analysis reveals that the groups differ regarding both the neural substrate and cognitive mechanisms behind the successful task solution (e.g. [34–38]).

## 6. The reorganization of elementary functions framework

Studies of the consequences of brain injury and post-traumatic cognitive recovery provide apparently contradictory results and theoretical dilemmas not unlike those found in blindsight.

At the most general level, one finds the contradiction between the concepts of ‘localization’ of various ‘functions’ (e.g. [39–41]) and functional ‘recovery’ as seen in animal models (e.g. [14,15,34–38]) and clinical studies (e.g. [42–46]). These two phenomena have a number of parallels to two of the prominent models of functional organization within the brain: the ‘modular’ theories proposed by, for instance, Fodor [47], Pinker [48] and Pylyshyn [49] (see review regarding ‘massive modularity’ in [50]), and the models emphasizing distributed, connectionist networks (e.g. [51–53]). Modularity emphasizes a strict functional localization, in which loss of the neural structure mediating that cognitive module leads to a cognitive impairment. On the basis of such a framework it is, however, hard to realize how a function may demonstrate a post-traumatic functional recovery (although some such models are less categorical and remain open to at least a certain degree of sparing/recovery, e.g. [49]). On the other hand, the connectionist framework makes it relatively easier to conceptualize the dynamic network reorganizations seen in post-traumatic cognitive rehabilitation—but can rarely account for the degree and specificity of initial trauma-associated impairments (e.g. [34,35]).

Perhaps less theoretically challenging, but important, are some of the issues mentioned in §3: the fact that the presence and nature of brain injury-associated symptoms appear to be dependent on details of the method of testing (in such a way that apparently cognitively similar variants of a given test may nevertheless provide completely contradictory results [14,15]); and the fact that even for identical injury to the same neural structure, the post-traumatic neural and cognitive reorganizations will depend on the task facing the individual [34,35].

Attempting to establish a comprehensive framework of the neural and cognitive mechanisms of post-traumatic functional recovery within problem solving, the reorganization of elementary functions (REF) framework was constructed [34–36,54,55]. The REF framework essentially describes a connectionist network in which, however, the ‘unit’ is not a neutral and functionally ‘indifferent neuron’, but advanced information processing modules called elementary functions. REF is thus able to account for both the localization and post-traumatic recovery of functions.

## 7. The reorganization of elementary functions and consciousness framework

As emphasized above, we see a number of striking similarities between some of the findings related to blindsight and the results achieved within studies of post-traumatic cognitive recovery of problem solving after focal brain injury. These similarities have prompted us to suggest a variation of the REF framework as a possible way to uncover neural correlates of consciousness (Mogensen & Overgaard [56]). We believe that this framework, called reorganization of elementary functions and consciousness (REF CON), can provide a comprehensive frame within which presently available data can be interpreted. Additionally, our theory will stimulate future research able to clarify many of the outstanding issues regarding both blindsight and the mechanisms of conscious perception more generally (see also [57]).

According to the original version of the REF framework [34,36,54,55] task solution, be it in the form of overt behaviour or mental representation, is achieved via two underlying levels: the lower level of the elementary functions and the level of the algorithmic strategies. Elementary functions perform basic information processing and are localized within restricted subdivisions of neural structures. By contrast, algorithmic strategies consist of numerous interacting elementary functions and are distributed in the sense that the neural substrate of an algorithmic strategy includes both the neural substrates of the individual elementary functions and the connections mediating the complicated interaction between these elementary functions. A given task solution is achieved via the computations of an algorithmic strategy. In a later elaboration of the REF framework, the level of algorithmic modules has been added [35]. Similar to an algorithmic strategy, an algorithmic module consists of a number of elementary functions and a pattern of interconnections between these elementary functions, computationally constituting a significantly higher level of information processing than that which is achieved by an individual elementary function. Algorithmic modules, however, differ from algorithmic strategies by not being able in themselves to mediate a task solution. The information processing of an algorithmic module constitutes a computational ‘subroutine’, which contributes to a number of algorithmic strategies. The major distinction between an algorithmic module and an algorithmic strategy is that the information processing of an algorithmic module is, in itself, insufficient to mediate a task solution. The distinction between an algorithmic module and an elementary function is that the information processing of an algorithmic module is more extensive and cognitively more specialized than that which is the case for an elementary function.

A given behavioural pattern or task solution may be achieved via different algorithmic strategies. Focal brain injury will deprive the individual of a substantial number of elementary functions and thereby all algorithmic strategies that depend upon those elementary functions. Thus, injury will lead to behavioural impairments of tasks previously achieved via activation of those algorithmic strategies (e.g. [34,35]). Subsequent training will, however, be able to establish novel algorithmic strategies (using preserved elementary functions). In addition, potentially the novel algorithmic strategies will be able to allow a task solution with a similar proficiency to what was seen pretraumatically. This ‘recovered’ task solution is, however, achieved via neural and cognitive mechanisms (‘strategies’) different from those present pretraumatically.

The REF CON framework is based on the same types of elementary functions, algorithmic modules and algorithmic strategies as the original REF framework. A crucial element within the mechanisms of REF CON is the perceptual elementary function (PEF) which differs from other elementary functions by receiving a more or less direct sensory input. ‘Ordinary’ elementary functions have no functional specificity reaching beyond their basic information processing (the association of an elementary function with a particular cognitive domain is purely determined by the dynamically changing input/output relationships of the elementary function). By contrast, a PEF is essentially ‘prewired’ to be associated with a sensory modality and thus with a perceptual analysis.

The PEFs are likely to be localized at subcortical as well as cortical levels and are the initial central processing steps for the incoming sensory information. It should be emphasized that the sensory information has been processed peripherally before reaching the PEFs of the brain—visual information has, for instance, undergone an extensive processing within the retina. Within the somatosensory system, some of the PEFs will be ‘modules’ within the somatotopic maps, while PEFs within the auditory modality include ‘modules’ within the tonotopic maps.

Any sensory input to the brain will result in the activation of, and information processing within, a number of PEFs. In most real-life situations, a sensory situation will be associated with the simultaneous activation of PEFs within several modalities.

The information processing of algorithmic strategies is shaped via backpropagation mechanisms emerging as a consequence of the situational feedback encountered when a pre-existing algorithmic strategy is activated and mediates behaviour. This experience-associated feedback and backpropagation-associated reprogramming shape the connectivity between elementary functions in a manner that can both modulate existing algorithmic strategies and create novel versions of such. The algorithmic modules emerge as a consequence of such modulations and reorganizations: when multiple algorithmic strategies share a network of elementary functions this network has become an algorithmic module. Through these kinds of mechanisms, PEFs as well as more ‘ordinary’ elementary functions are combined into networks which constitute the perceptual algorithmic modules. These networks (perceptual algorithmic modules) are not in themselves able to either direct behavioural activity or form the basis of conscious representations. They are, however, crucial elements in the process leading to behavioural control as well as conscious awareness.

The perceptual algorithmic modules are organized in a hierarchical manner reaching from relatively low-level modules (situated close to the initial sensory input) to algorithmic modules which become incorporated in a special type of algorithmic strategy termed the situational algorithmic strategy (SAS). When incorporated in the SAS, a perceptual algorithmic module becomes able to direct behaviour and potentially reach conscious awareness.

**Figure 1.**
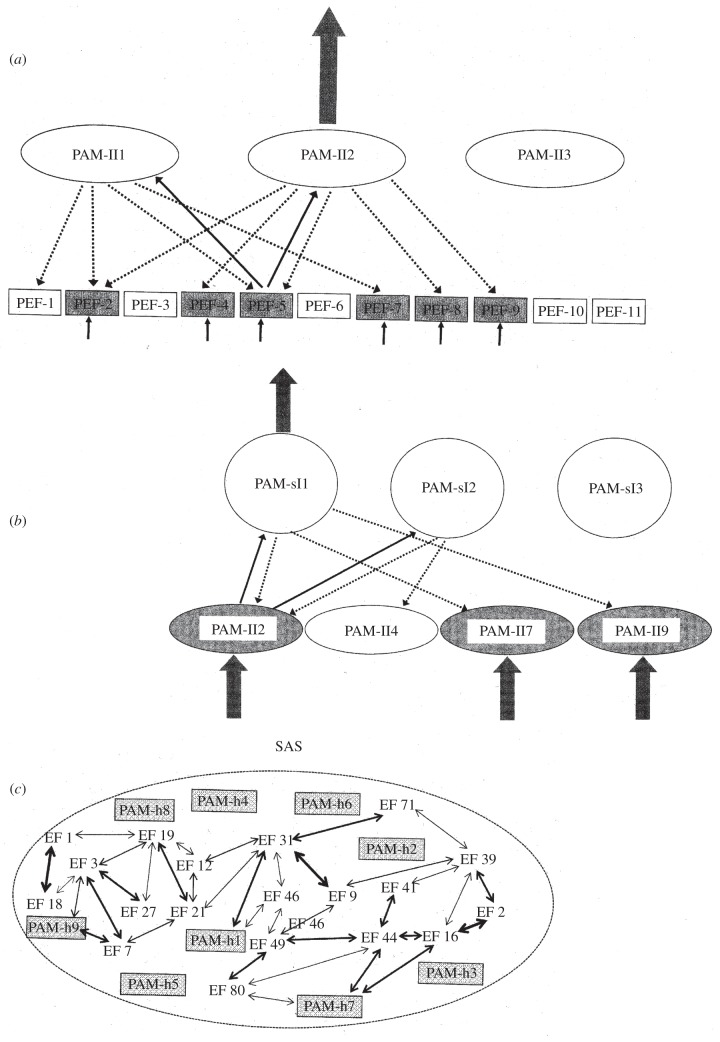
Schematic and simplified representation of central aspects of the REF CON framework. (*a*) Sensory activation of PEFs (shaded PEFs are activated) leads to activation of perceptual algorithmic modules of the lowest level (PAM-IIs). Activated perceptual algorithmic modules ‘interrogate’ PEFs and the perceptual algorithmic module with the best correspondence between activated and interrogated PEFs receives full activation. (*b*) Fully activated perceptual algorithmic modules of the lowest level activate a number of perceptual algorithmic modules of the second level (PAM-sIs) which in turn interrogate activated as well as non-activated perceptual algorithmic modules of the lowest level. Again, the perceptual algorithmic module with the best correspondence between the interrogated and activated entities at the immediately lower level receives full activation. (*c*) The SAS, including perceptual algorithmic modules of the highest level (PAM-hs): some are left unintegrated into the SAS, while some are integrated at a variety of levels. For further details, see §7.

Whenever a pattern of activation of PEF occurs, the activated elementary functions will automatically lead to a degree of activation of a number of perceptual algorithmic modules of the lowest level of this hierarchy (as shown in figure 1*a*). Every PEF is an element within a huge number of perceptual elementary modules of the lowest level. In addition, all of these modules will receive a degree of activation when such a PEF is activated. The degree of activation of a given perceptual elementary module of the lowest level will depend heavily upon how many of its constituent PEFs are active at that moment in time. For each of the, in this way, activated perceptual elementary modules, the activation initiates a testing of the degree of coherence within the activation of its constituent elementary functions. Each of the activated perceptual elementary modules will ‘interrogate’ all of its constituent PEFs as to their degree of current activation. In the case of a high degree of ‘mismatch’ between the pattern of interrogated and activated PEFs, respectively, the algorithmic module in question will not reach a significant level of activation. If, however, there is a reasonable level of congruity of the current pattern of PEF-activation and the pattern ‘expected’ by the algorithmic module in question, a full activation of that module is likely to occur. The degree of activation of perceptual algorithmic modules in a given situation is also a result of mutual competition and inhibition between related perceptual algorithmic modules at this lowest level. It should be remembered that the networks constituting the perceptual algorithmic modules at a given level are heavily interconnected and partly overlapping.

Within the hierarchically organized perceptual algorithmic modules, the modules at the lowest level are constituent elements within the perceptual elementary modules of the next level. Activated modules at the lowest level will lead to a degree of activation of a number of perceptual elementary modules at the next level (as illustrated in figure 1*b*). The process determining which of these activated modules at the second lowest level will eventually reach full activation is essentially similar to what was described with respect to the selection of the perceptual elementary modules of the lowest level. The algorithmic modules at the second lowest level which are best able to ‘account for’ the pattern of activation of the lowest level algorithmic modules will reach a level of full activation, thereby activating a number of perceptual algorithmic modules at the next level which will then enter into a mutual competition as occurred at the lower levels.

The number of levels of perceptual algorithmic modules through which the perceptual analysis is conducted is determined by multiple factors. The type of sensory information to be analysed is a factor. But the level of experience of the individual, as well as the potential presence of injury or dysfunction, are other factors impacting upon the hierarchy of such algorithmic modules. But no matter what the condition, the ongoing activation of perceptual algorithmic modules of constantly higher levels will continue until the activated algorithmic module does not feed into any higher level of perceptual algorithmic modules. This may be when an essentially ‘full analysis’ of the sensory input has been achieved. Or it may be due to levels of algorithmic modules being unavailable because of the lack of experience with the sensory information in question, or injury/dysfunction within the perceptual systems. Regardless of the reasons, the ultimate (i.e. highest available) level of perceptual algorithmic modules activated in a given perceptual process will eventually be reached, and represent under the current circumstances the most advanced perceptual analysis available.

It should be noted that at all the levels of perceptual algorithmic modules, the selected module represents the ‘best available fit’ rather than a 100% fit to the activation pattern of the immediately lower level. Thus, the current model includes contributions from both bottom-up and top-down processes, top-down processes not being in the least determined by the pre-existing connectivity within perceptual algorithmic modules of various levels. As pre-existing connectivity is shaped by experience, the bottom-up/top-down interplay predicts that the contents of experience in many cases will be different between individuals even under the same circumstances.

The progression through which the sensory input causes the selection of perceptual algorithmic modules of the lowest level and subsequently higher levels, is a constant interplay between ‘forward’ activation and feedback activation interrogating the original activation pattern at the immediately lower level. Such a process agrees well with the results of Garrido *et al*. [58]. In an analysis of long-latency event-related potentials (ERPs), Garrido *et al*. [58] found that such responses are best explained by an interaction between the activation of forward and backward connections within the systems in question. This is a different perspective on, but in agreement with, findings that visual experience is related to feedback signals to primary sensory areas [59].

Only the perceptual algorithmic modules of the highest level of the above-described hierarchy are (potentially) available to the regulation of behaviour and/or conscious experience. With well-known material and intact perceptual systems, an individual will thus only experience the relatively ‘completely analysed’ percept. All previous steps of the perceptual analysis will remain unavailable to behavioural control and/or subjective awareness. If, however, injury or dysfunction prevents parts of the perceptual analysis, what is available to behaviour and/or consciousness can be of a more ‘basic’ and potentially fragmented nature, simply because what before was a ‘primitive’ is now the best possible ‘highest level representation’. A similar situation may occur if the stimulus material is unknown or presented under such atypical circumstances that the higher levels of analysis are unavailable.

The analysed sensory input represented by the perceptual algorithmic modules of the highest level is in such a form that it is not available as either the basis for action or conscious awareness. To reach such a level, it will have to become part of an algorithmic strategy. In the case of perceptual algorithmic modules of the highest level, they will have to be integrated into a special type of algorithmic strategy called the SAS (figure 1*c*).

The SAS is a highly dynamic algorithmic strategy that reflects the current status of the individual. The defining feature of the SAS is that it combines elements within sensory/perceptual, motor/behavioural, motivational/planning and other systems. Some elements within this network are perceptual algorithmic modules of the highest level representing the external world. Other elements within the SAS reflect aspects of the internal world, as well as the current goal situation in terms of what objectives the individual is attempting and/or intending to achieve. Additionally, the SAS contains patterns reflecting the ongoing behavioural activities. The SAS is in a constant state of dynamic flux, being modified according to changing motives, goals, emotional fluctuations as well as sensory stimulation, thereby being even more dynamic and constantly modified than is the case with other algorithmic strategies.

If relevant to the current situation of the individual, the perceptual algorithmic modules of the highest level will be integrated into the SAS. Once integrated in this network, a perceptual algorithmic module will be able to influence the behaviour of the individual.

The integration of a perceptual algorithmic module into the SAS can take numerous forms. Any level of integration allows the perceptual algorithmic module to influence the ongoing and planned behaviour of the individual. However, only more elaborate integration and extensive connections to various other elements within the SAS allow the perceptual algorithmic module in question to influence the conscious awareness of the person. In other words, whether the information associated with a perceptual algorithmic module will be consciously available is determined by the degree and pattern of connectivity between, on the one hand, the perceptual algorithmic module and, on the other hand, other components of the SAS.

## 8. Reorganization of elementary functions and consciousness and the mechanisms of consciousness

The determining factor regarding whether or not the content of a perceptual algorithmic module is conscious is not the involvement of any particular elementary function but rather a pattern of computational interactions between the perceptual algorithmic module in question and other parts of the SAS. Such a pattern is influenced (among other factors) by the task currently facing the individual. Thus, for instance, different patterns of interactions between elementary functions will be associated with situations calling for a direct conscious perception or a situation calling for a metaconscious approach, respectively. These connectionistic differences are primary factors determining the differences between results obtained under such circumstances (e.g. [60]).

A crucial factor in determining whether and to what extent a perceptual algorithmic module is integrated into the SAS, and thereby is consciously experienced, is how relevant the percept represented by that algorithmic module is to the broadly defined actions of the individual. Such actions may be in the form of overt behaviour, but could also be ‘inner action’ in the form of thought processes.

As mentioned as a basic consideration for the REF CON framework, conscious experience relates to the ‘most analysed’ information from a functional perspective, i.e. the information that is most relevant for or available to action. Thus, REF CON would not predict any particular brain area as being necessary and sufficient for conscious experience. Perceptual algorithmic modules of the highest level integrated into the SAS are experienced regardless of which elementary functions are involved. For this reason, identifying a particular neural process associated with a given conscious experience does not lead to the conclusion that the absence of this neural process in other subjects or animals means absence of consciousness.

The incorporation of perceptual algorithmic modules of the highest level into the SAS is a situational and short-term process which has to be mediated by neural processes able to manifest themselves with an extremely short latency. The types of synaptic and general structural plasticity involved in more long-term reorganizations of algorithmic strategies cannot mediate such a situational process.

## 9. Training of conscious experience

In blindsight, the fact that visual information is typically not used spontaneously but can only become a factor in the behavioural control when the subject is prompted or given very specific instructions (e.g. [8,11]), appears to indicate that in these cases, the relevant perceptual algorithmic modules can only become integrated into the SAS when an external instruction has modified the SAS to be receptive to such an integration.

Normally, a perceptual algorithmic module of the highest level will become integrated into the SAS, and thereby become available to the control of behaviour, without any deliberate effort from the individual. That is, provided what is represented by the algorithmic module in question is relevant to the current goals of the individual. As mentioned in §2, the situation in blindsight appears to be somewhat different. The information potentially available in blindsight is not spontaneously used in behaviour. For blindsight to be demonstrated, external instructions are needed in combination with a deliberate effort on the part of the subject (e.g. [8]).

The somewhat rudimentarily analysed visual information available in blindsight is, according to the REF CON framework, a reflection of a process in which the visual analysis has progressed through only a relatively low number of levels of perceptual algorithmic modules. Therefore, the analysis will reach its most advanced level, the highest level, at a comparably earlier stage of analysis than that which is normally seen. Although these earlier levels can become integrated into the SAS (and thereby made available to behavioural control), such a process requires an unusual degree of top-down control and effort.

As previously described, the degree to which an individual is conscious of a stimulus depends on the degree and pattern of connectivity between, on the one hand, the perceptual algorithmic module of the highest level representing that stimulus and, on the other hand, the rest of SAS. The more elaborate this connectivity is, the higher the level of conscious awareness of the stimulus will be.

The studies in which a less dichotomous and more graded evaluation of conscious awareness (for instance, using the PAS; e.g. [13]) has been applied do, however, indicate that at least a certain degree of conscious awareness can be demonstrated whenever a stimulus is able to direct behaviour. On such a background, it seems that whenever a perceptual algorithmic module is integrated into the SAS, it is not only available to behavioural control but also to conscious awareness, at least at a rudimentary level. The degree of conscious awareness will increase with more elaborate connectivity and thereby more elaborate information processing.

In contrast to these immediate processes of the actual incorporation of the perceptual algorithmic modules into the SAS, the mechanisms achieving the incorporation of perceptual algorithmic modules into the SAS can be modified by training. In addition, these modifications are likely to be mediated by neuroplastic processes similar to those achieved via the backpropagation mechanisms reorganizing other algorithmic modules (e.g. [35]). Using the metacontrast masking experimental set-up, it has been demonstrated that training can increase the utilization and subjective awareness of stimuli which were originally neither able to influence behaviour nor to be consciously perceived (e.g. [61]). Thus, training can influence whether and even how perceptual algorithmic modules are integrated into the SAS. These training effects are likely to have at least some mechanisms in common with the demonstrated effects of training in the case of blindsight. In a forced choice procedure, performance can be improved by training in both monkeys subjected to bilateral ablation of the primary visual cortex (V1) [62,63] and patients demonstrating blindsight (e.g. [64–70]). In blindsight patients, training may not only improve the behavioural performance, but also the subjective awareness (e.g. [71]). In intact individuals presented with subliminal stimuli, the subjective awareness of these stimuli appears to be able to be increased by training procedures (e.g. [61,72]).

The effect of ‘training’ and experience can also be demonstrated in contexts which are more likely to cause modifications within perceptual algorithmic modules (of various levels). If part of the somatosensory input is lacking, thereby leaving part of the somatotopic representation ‘vacant’, a reorganization of this representation will occur. If, for instance, a hand is amputated, the contralateral somatosensory representation will undergo a change in which the two representations (the face and arm, respectively) neighbouring the now ‘vacant’ hand area will ‘grow’ and eventually completely cannibalize the area previously representing the now amputated hand (e.g. [73–75]). Thus, within the somatotopic representation, the area previously receiving input from the now absent hand will become activated by input from body-parts previously unable to activate this part of the somatotopic map. In terms of the present model, PEFs which were originally activated by input from the hand will now become activated by input from, for instance, the face. Although the actual input activating the previous somatosensory hand-representation is now originating from another body-part, the individual may for a period of time still experience input to the brain region in question as originating from the now absent hand (e.g. [76,77]). Only at a later point in time, when the individual has undergone additional ‘training’ in the form of daily experiences, will the sensory input activating the previous hand area be experienced as actually originating from its novel source (rather than from the amputated hand). In terms of the present framework, the mechanisms of this process are that while PEFs of the previous hand area quickly undergo changes activating them by input from face and arm, respectively, the perceptual algorithmic modules which process the activation of these PEFs are not reorganized as quickly. For a period of time, these perceptual algorithmic modules are still organized in ways which ‘assume’ the input to the hand-PEFs still to originate from the hand. Subsequent experiences, backpropagation mechanisms and reorganizations are necessary for these algorithmic modules to allow the individual to consciously experience activation of the previous hand area as representing input from the body areas from where the stimulation now actually originates.

## 10. Perception shaped by learning

A somewhat different example which nevertheless may reflect related processes can be found in the patient SB reported by Gregory & Wallace [78] and Gregory [79]. SB grew up virtually blind. Aged 52 years, SB received corneal grafts to both eyes and gained the ability to see. When postoperatively first experiencing vision, SB appeared to be almost exclusively able to visually perceive what had been available to tactile exploration. In a striking example, SB when drawing a bus (offered to his visual inspection) included a type of wheel which was no longer used on buses. SB had, however, as a child (using tactile exploration) experienced that type of wheel. When later again tasked with drawing a visually presented bus, SB now drew a wheel which was identical to the one actually present. Apparently, the visual information offered to SB was originally analysed through perceptual algorithmic modules which had primarily been constructed and structured using tactile information (while SB was blind). Only through a prolonged period of visual experiences were the perceptual algorithmic modules of SB reorganized into a structure representing the (visual) world of the present day.

As previously mentioned, perceptual algorithmic modules of the lowest level are under normal circumstances likely to receive activation from PEFs associated with multiple sensory modalities. Thus, within the framework of REF CON, multimodal perceptual phenomena such as the McGurk illusion [80] can easily be accounted for. The McGurk illusion (the fact that auditory perception of syllables is modified by simultaneously watching a face pronouncing a different syllable) reflects the fact that even when consciously perceived as a purely auditory process, auditory perception of spoken language is in reality a multimodal process whenever relevant visual information is available. Some of the neural mechanisms associated with this multimodality are presently being analysed (e.g. [81]) and such an analysis may provide cues regarding the neural networks mediating the associated perceptual algorithmic modules.

Since the perceptual algorithmic modules involved in various types of perceptual analysis are constantly subjected to use-associated feedback and potentially backpropagation-provoked reorganizations, REF CON predicts that plastic processes are able to allow not only reorganizations within a given modality (e.g. [76,77]) but also reorganizations reaching across sensory modalities, as seen in various types of cross-modal plasticity (e.g. [82]). Mancuso *et al*. [83] emphasize that according to most traditional views of colour perception it is surprising that gene therapy is able to eliminate colour blindness in adult primates. According to most prevailing theories, the mechanisms of colour perception become ‘hard-wired’ in early development and should not be able to use an adult insertion of a peripheral analysis of colour. REF CON, on the other hand, includes a number of dynamically reorganizable levels (the levels of perceptual algorithmic modules) between the sensory input to the brain and the mechanisms of the actual perceptual experience. Thus, REF CON can more easily account for such an ability to use an adult's age acquired ability to receive colour-coded input.

## 11. Studies of the neural correlate(s) of consciousness

Most current theories about the neural correlate(s) of consciousness (NCC) suggest some specific neural region as necessary, or at least sufficient, for subjective experience. For instance, Logothetis and colleagues have found that inferior temporal regions along the ventral projection streams from primary visual cortex are active in relation to the dominant percept during binocular rivalry [84]. On this background, they suggest these regions as an interesting candidate for an NCC. Rees *et al*. [85] review a number of experiments and argue that activations cluster around parietal and prefrontal sites when subjects experience changes in bistable figures, binocular rivalry, and when they become consciously aware of changes in complex visual scenes. Lamme [59], however, suggests that we can only be conscious if there are feedback loops to primary visual cortex based on, primarily, ERP experiments showing that visual information can reach even prefrontal areas without accompanying conscious experiences.

There are more proposals for NCC candidates than those which can be mentioned here. The few examples above aim to show that such candidates are rather different, and cover all cortical lobes. One difficulty for all such proposals is that they can only account for a limited amount of findings, and they are forced to explain why other experiments, with sometimes small methodological variations, give rise to very different findings.

One advantage of the REF CON framework is that it does not presuppose that there is any ‘consciousness region’ in the brain. Rather, it would predict that the level of cognitive representation, related to particular algorithmic strategy, correlates with consciousness. Even though the elementary parts of the model are expected to be localized in different regions, there is not one particular constellation of functions in one particular kind of strategy that is expected to be ‘more conscious’ than other possible constellations. For the same reason, REF CON has no principal problems explaining data that in the light of other theories look like conflicting data.

The REF CON framework shares more similarities with the relatively few computational theories of consciousness. According to Tononi [86], consciousness is identical to ‘integrated information’, i.e. consciousness is the property of a physical system, and its quantity can be specified mathematically. Crick & Koch [87] argued that there might be a common neural mechanism behind ‘the binding together’ of information that is otherwise separately processed in the brain and mechanisms of consciousness. Therefore, they argued that synchronous activity in the ‘gamma range’ is causally involved in consciousness. Although those theories share with REF CON the idea that consciousness is not directly related to particular regions in the brain, they are, obviously, based on different assumptions about which properties to associate with consciousness. REF CON's association between consciousness and ‘level of representation’ is based on empirical data, as argued here and in §§9 and 10.

## 12. Conclusion

The REF CON framework is a theoretical proposal aimed at resolving seemingly contradictory evidence in blindsight and subliminal perception research. The model has a general scope, and is therefore applicable to any aspect of consciousness research without modifying the principal ideas. One advantage of REF CON is its ability to combine seemingly conflicting evidence in one, hierarchical organized model with several levels. Conflicting data on blindsight and subliminal perception can be resolved essentially by arguing that conscious experience is not in opposition to functional aspects of mind, but rather, an intrinsic part. Thus, the model opposes the idea that we can have ‘access consciousness’ about something of which we are ‘phenomenally unconscious’. Methodological developments in both fields have questioned the classical dichotomy between ‘on’ and ‘off’ versions of mental states, and show that vague perceptions may underlie what seems to be ‘fully unconscious perception’. The neuroscientific side of REF CON suggests a combination of organizational principles that are otherwise often presented as opposite ideas, namely the idea of functional localization, and the idea of a distributed, connectionist system. The model essentially suggests that no particular brain area or process is itself associated with consciousness, but that information analysed to a given ‘highest level’ and then integrated into the SAS is experienced. The model obviously predicts close correlations between information that is analysed to this degree and experience. But denies that one level of description can be said to create another.

## Funding statement

M.O. was funded by a Starting Grant from the European Research Council, and J.M. by a grant from the Danish Council for Independent Research.
